# Vocal membranes lower the phonation threshold pressure in rhesus macaques (*Macaca mulatta*)

**DOI:** 10.1098/rsos.250243

**Published:** 2025-06-25

**Authors:** Mayuka Kanaya, Rintaro Miyazaki, Tomoki Yoshitani, Takeshi Nishimura, Isao T. Tokuda

**Affiliations:** ^1^ Ritsumeikan University, Graduate School of Science and Engineering, Kusatsu, Shiga, Japan; ^2^ The University of Osaka, Graduate School of Human Sciences, Suita, Osaka Prefecture, Japan

**Keywords:** bioacoustics, vocal membrane, macaques, animal vocalization, computational model

## Abstract

The vocal membrane, an extended part of the vocal fold, is present in a broad range of species including non-human primates. Its contribution to animal vocalizations has long been of interest. A theoretical study (Mergell P, Fitch T, Herzel H. 1999 Modeling the role of nonhuman vocal membranes in phonation. *J. Acoust. Soc. Am.* **105**, 2020–2028. (https://doi.org/10.1121/1.424994)) predicted that vocal membranes enhance vocal efficiency by lowering the phonation threshold pressure. To test this, excised larynx experiments were conducted on rhesus macaques (*Macaca mulatta*). Comparisons before and after surgical removal of the vocal membranes showed that the phonation threshold pressure was indeed lower—and vocal efficiency higher—when the vocal membranes were present. Most experiments exhibited periodic oscillations of the vocal folds and/or membranes, while some showed irregular broadband oscillations potentially indicative of chaos. A computational model representing the vocal membrane as a dynamic, reed-like plate reproduced both periodic and irregular oscillations, depending on parameter settings such as the damping ratio. These simulations suggest that transitions between different regimes can arise from individual anatomical or physiological variation. Although this pilot study is based on two macaque larynges, the results support the idea that vocal membranes may contribute to vocal efficiency and dynamic variability, potentially enabling louder calls with less pulmonary effort.

## 1. Introduction

The vocal membrane (or vocal lip), i.e. an appendage extending from the vocal fold, is present in a range of species including bats and primates [1–4]. It is attached to a supero-medial portion of the vocal fold and extends superiorly along the medial surface of the vocal fold. This anatomical structure suggests that adduction of the vocal folds may move the bilateral vocal membranes towards the glottal midline, inducing their oscillations during vocal source generation. From the viewpoint of animal vocalizations, understanding the role of the vocal membrane has been a matter of great interest. Acoustic functions of the vocal membrane are, however, yet to be fully understood. Mergell *et al*. [5] developed a theoretical model of the vocal membrane and showed that it can make animal vocalizations more efficient by lowering the phonation threshold pressure. This effect is pronounced when the vocal membranes are tilted at a certain angle and their oscillation frequencies are relatively high. It was also suggested that this additional tissue may extend the regime of chaotic oscillations. The theoretical study was then followed by animal experiments. Brown *et al.* [6] carried out an excised larynx experiment on a squirrel monkey (*Saimiri boliviensis*). Although the observed ridge on the superior part of the vocal fold did not resemble a typical shape of the vocal membrane, they reported that irregular calls with flat spectral structures were occasionally observed. Zhang *et al.* [7] reported from excised larynx experiments of common marmosets (*Callithrix jacchus*) that, through laryngeal development, the sound source switched from vocal folds to vocal membranes, which produced louder vocalizations with a higher efficiency. Compared with the infant larynx, in which the vocal membranes are yet to be developed, the adult larynx with vocal membranes realized vocalizations with a significantly reduced subglottal pressure, supporting the theoretical prediction of Mergell *et al.* [5]. Nishimura *et al.* [4] observed laryngeal dynamics of primate species *in vivo* and found that the vocal lips gave rise to sole oscillations or co-oscillations with the vocal folds to contribute to the generation of the source sounds. Moreover, they reported that a nonlinear interaction between the vocal folds and membranes can sometimes lead to voice instabilities. Håkansson *et al.* [8] showed from excised larynx experiments of bats (*Myotis daubentonii*) that only the vocal membranes vibrated at a frequency range from 10 to 20 kHz, where no vibration of the vocal folds was observed.

The present study focuses on the theoretical prediction of Mergell *et al.* [5], since it presents one of the clear advantages of using the vocal membranes in animal vocalizations. We examine the hypothesis that the vocal membrane lowers the phonation threshold pressure. As a related study, Kanaya *et al.* [9] constructed a synthetic model of the vocal membrane and showed that its onset pressure was decreased in the physical experiment. Although the synthetic model well approximated the geometry and biomechanical properties of the real vocal membranes, it did not represent the exact physiological feature of the animals. In the present study, we used an excised larynx set-up to examine the hypothesis. As the subject species, rhesus macaque (*Macaca mulatta*) was examined.

It has been known that visual information is used extensively in primate communication [10], including gestures and facial expressions in macaques [11–14]. In addition to visual communication, vocal communication also plays an important role for macaques, especially in deep forests where visual information is not available [15,16]. It is therefore of value to investigate the mechanism of producing various vocal patterns and how the vocal membranes contribute to them in macaques.

In the present study, the phonation onset pressure was measured by injecting an airflow into the macaque larynx. As a control condition, the same experiment was repeated after the vocal membranes were surgically removed from the vocal folds. Oscillation patterns of the vocal membranes were also observed by a high-speed filming technique. Our interest is whether the phonation onset pressure is lowered in the presence of the vocal membranes. To understand the oscillation mechanism of the vocal membranes and to seek for its influence on the phonation onset pressure, a computational model that reproduces qualitative features of the experimental observations was also simulated.

## 2. Material and methods

### 2.1. Excised larynx experiments

From three fresh cadavers of adult female macaques (nos. 1, 2 and 3), the laryngeal samples were extracted, flash-frozen in liquid nitrogen, and stored at $-80^{\circ }$C. Quick freezing has been reported to be efficient in preserving laryngeal tissues used for excised larynx experiments [17]. The frozen samples were thawed shortly before the excised larynx experiments, which were performed under two conditions: (A) in the presence of the vocal membranes and (B) in the absence of the vocal membranes. Under both conditions, the individual larynx was mounted on a vertical tracheal tube. The flow rate of humid air (approx. 37°C; 100% relative humidity) that comes from an air pump (SilentAirCompressor Sc820, Hitachi Koki Co., Ltd., Tokyo, Japan) was controlled by a pressure regulator (10 202U, Fairchild, Winston-Salem, NC) and a digital mass flow controller (CMQ-V, Azbil, Santa Clara, CA). The flow controller was regulated by a computer (MacBook Pro, 2.8 GHz Intel Core i7, C02H30R8DV17) through a LabJack interface (U6, LabJack). To induce the vocal fold vibrations, the glottal air space was narrowed by manually adducting the arytenoid cartilages. Once the adjustment was made, positions of the arytenoid cartilages were fixed by a surgical suture (detailed in the electronic supplementary material, figure S1). This configuration was used commonly for the two conditions (A) and (B). Namely, without changing the level of adduction, the vocal membranes were removed manually by the surgical scalpel to switch the condition from (A) to (B).

In condition (A), two oscillation patterns were observed: (A1) the vocal folds did not show a strong vibration and only the vocal membranes oscillated, and (A2) both vocal folds and vocal membranes oscillated simultaneously. The oscillation pattern (A1) was observed when the upper parts of the vocal folds, i.e. vocal membranes, were strongly adducted, while the lower parts of the vocal folds were not much adducted. The oscillation pattern (A2) was observed when both upper and lower parts of the vocal folds were adducted. To induce the oscillation pattern (A1), the arytenoid cartilage was pressed by the experimenter’s hands or micromanipulators (BMF-1, BEX Co. Ltd., Tokyo, Japan), which rotated the vocal process of the arytenoid towards the inferior direction. By this rotational movement, the left and right vocal membranes attached to the body part of the arytenoid were located close to each other, while the lower parts of the left and right vocal folds attached to the vocal process were set apart from each other (detailed in the electronic supplementary material, figure S1).

The dynamics of the vocal folds and the vocal membranes was monitored using a borescope with a view angle of 70${,}^{\circ }$ (BAL-72718HT, Shodensha, Osaka, Japan) attached to a high-speed video camera (Fastcam Nova S6, Photron, Tokyo, Japan; sampling: 10000 FPS, shutter speed: 125 000 s^−1^). The acoustic sound and the sound pressure level (SPL) were measured by an omnidirectional microphone (Type 4192, Nexus conditioning amplifier, Brüel and Kjaer, Tokyo, Japan) and a sound level meter (Type 2250-A, Brüel and Kjaer), respectively, both located 15 cm from the larynx. The subglottal pressure was monitored using a pressure transducer (differential pressure transducer, PDS 70GA, Kyowa, Osaka, Japan; signal conditioner, CDV 700A, Kyowa), which was mounted flush on the inner wall of the tracheal tube, 2 cm upstream of the excised larynx. All signals were stored into a digital recorder (controller, PXIe-8840, National Instruments; Input/output card, BNC−2110, National Instruments; Software, Labview, National Instruments, Austin, TX, USA) with a sampling frequency of 12.5 kHz.

Before the measurement of the phonation onset pressure, a preliminary experiment was conducted to detect the onset airflow at which the vocal folds started to oscillate. Then, the phonation onset pressure was measured by slowly increasing the flow rate from 0 l min^−1^ to a maximal value in 3 s. The maximal airflow, which was set to be about 1.5 times of the pre-measured onset airflow, ranged between 16.0 l min^−1^ and 32.2 l m^−1^ for macaque no. 1 and between 16.0 l min^−1^ and 28.3 l min^−1^ for macaque no. 2. The phonation *onset* was detected at the pressure, where the oscillation amplitude exceeded a threshold value of 150 Pa. After the onset point, self-sustained oscillations of the larynx continued to be measured for 4 s. Next, the flow rate was decreased to 0 l min^−1^ in 3 s and the phonation *offset* was detected when the oscillation amplitude became less than the threshold.

For each of the three oscillation patterns (A1), (A2) and (B), the onset and offset pressures were measured 10 times and their average and the standard deviation were computed as the results.

### 2.2. Data analysis

From the microphone signals, which recorded the self-sustained oscillations of the excised larynges for more than 4 s, the fundamental frequencies $f_{\text{o}}$ were computed by Praat software (www.praat.org, v. 6.0.50). The vocal efficiencies were calculated as $E=10\log_{10}\frac{4\pi r^{2}I}{UP_{s}}$, where r represents distance from the excised larynx to the microphone, $I=10^{\text{(SPL/10)-12}}$ (W m^−2^) is the sound intensity obtained by using the sound pressure level SPL (dB), U is the flow rate (m^3^ s^−1^) and $P_{s}$ (kg m^−1^ s^−2^) is the subglottal pressure [18,19]. To quantify the level of complexity in the laryngeal vibrations, permutation entropies [20,21] as well as spectral entropies [22] were further computed from the microphone signals by the MATLAB (R2021b; MathWorks, Natick, MA, USA). These quantities have been widely applied to real data as a measure of complexity. In the permutation entropy, the time series was converted into symbolic sequences, and the information entropy of the sequential patterns was computed [20,21]. In the spectral entropy [22], the complexity was measured in a frequency domain. Namely, the *Fourier* transform of a time series was treated as a probability distribution of frequency components and its information entropy was calculated.

Compared with the traditional methods of detecting chaos in time series by, e.g. estimating Lyapunov exponents or correlation dimensions [23,24], the permutation entropy is very robust against noise, because it is based on a coarse-graining of the measured time series. Although the permutation entropy provides one of the most reliable methods to detect chaos in time series, it can detect complexity not only in chaotic signals but also in noise signals [20,21]. When complexity is detected in the present excised larynx data, our analysis does not exclude the possibility that the complexity comes from a stochastic factor. In that sense, the results should be treated with care. It has been known, however, that the laryngeal dynamics is governed by a relatively low-dimensional deterministic system, in which noise does not usually play a major role in causing irregular oscillations [25–28]. We, therefore, consider it is reasonable to apply the permutation entropy analysis to detect chaos in the present data.

To examine the statistical difference between three oscillation patterns (A1), (A2) and (B), a one-way ANOVA was performed. Since the individual variability is large in the present animal study, the effect of the vocal membrane was evaluated within the same individual. When a significant difference was detected for the three patterns (p<0.05), multiple comparison (Tukey’s honest significant difference, HSD) was carried out to examine the difference between the individual conditions.

### 2.3. Computational model

In the studies of voice production, computational models have played an important role in elucidating the mechanism of vocal fold oscillations [29–31]. To understand the oscillation mechanism of the vocal membranes and to seek for its influence on the phonation onset pressure, a computational model was simulated. Our model is based on the one proposed by Neubauer [32]. Compared with the previous model, in which the vocal membrane was described as a static object and its angle does not change in time [5], the vocal membranes move in time in the present model. Despite its simple structure, it may reproduce qualitative features of the observed experiment. Figure 5a illustrates the model configuration. The vocal folds are represented by a two-mass model, composed of lower and upper masses ($m_{1}$,$m_{2}$). On the upper mass, the vocal membrane is attached as a reed-like plate that can vibrate. In the model construction, the following is assumed:

(i) left and right movements are symmetric for the vocal folds and the vocal membranes [29];
(ii) influences of the sub- and supra-glottal resonances are neglected [30];
(iii) below the narrowest part of the glottis, the intra-glottal pressure is governed by Bernoulli’s law [33]; and
(iv) collision forces, which could arise during the contact of the left and right vocal membranes, are neglected [32].

The model equations read:


(2.1)m1x¨1+r1x˙1+k1x1+kc(x1−x2)−Θ(−a1)c1|a2|2L=Ld1P1,(m2+m3)x¨2+r2x˙2+k2x2+kc(x2−x1)−12m3d3(θ¨cosθa−θ˙2sinθa)(2.2)−Θ(−a2)c2|a2|2L=Ld2P2+L∫0d3cosθaP3(y)dy,(2.3)13m3d32θ¨+r3θ˙+k3(θ+ηθ3)−12m3d3x¨2cosθa=−Lcos2θa∫0d3cosθayP3(y)dy.

The dynamical variables $x_{i}(t)$ represent displacements of the two masses (i=1: lower mass, i=2: upper mass), while $\theta_{a}(t)=\theta_{0}+\theta (t)$ represents the angle of the vocal membrane ($\theta_{0}$: pre-phonatory angle, θ: deviation from $\theta_{0}$). The constant parameters $m_{i}$, $r_{i}$, $k_{i}$, $c_{i}$, $d_{i}$ represent weight, damping, stiffness, collision stiffness and thickness of the two masses (i=1,2) and the vocal membrane (i=3), respectively, while the parameter $k_{\text{c}}$ represents a coupling between the two masses. For the stiffness of the vocal membrane, cubic nonlinearity was considered with a constant η. The collision function is approximated as Θ(x)=0 (x≤0); Θ(x)=tanh⁡(1000x) (0<x). The glottal areas of the lower and upper masses are given by $a_{i}=a_{0i}+2Lx_{i}$ (i=1,2), where $a_{0i}$ is the prephonatory opening area and L is the glottal length. The area function along the vocal membrane is given by $a_{3}(y)=a_{2}-2L\;y\;\tan\theta_{a}$ ($0\;\leq \;y\;\leq d_{3}\;\cos\theta_{a}$), while the area at the tip of the vocal membrane becomes $a_{\text{vm}}=a_{3}(d_{3}\cos\theta_{a})=a_{2}-2Ld_{3}\sin\theta_{a}$. The opening area of the vocal folds is determined as $a_{vf}=\min(a_{1},a_{2})$, while the narrowest opening area of the glottis is given by $a_{\text{min}}=\min(a_{1},a_{2},a_{\text{vm}})$. The pressures $P_{1}$, $P_{2}$, $P_{3}$, which act on the right-hand side of equations (2.1)–(2.3), and the glottal volume flow are described by Bernoulli’s law as:


(2.4)P1=Ps[1−(aminΘ(amin)a1)2]Θ(a1),(2.5)P2=Ps[1−(aminΘ(amin)a2)2]Θ(a1)Θ(a2)Θ(a1−avm)Θ(a2−avm),(2.6)P3(y)=Ps[1−(aminΘ(amin)a3(y))2]Θ(a1)Θ(a2)Θ(a1−avm)Θ(a2−avm),(2.7)u=2PsρaminΘ(amin),

where $P_{\text{s}}$ represents the subglottal pressure and ρ is the air density.

To simulate the dynamics of the vocal membranes, parameter values of the vocal fold were scaled from the standard ones [30,32] as: $m_{1}=0.781$ g, $m_{2}=0.0156$ g, $d_{1}=0.1$ cm, $d_{2}=0.02$ cm $k_{1}=0.2$ g ms^−2^, $k_{2}=0.02$ g ms^−2^, $k_{c}=0.0625$ g ms^−2^, η=1, $c_{1}=3k_{1}$, $c_{2}=3k_{2}$, $r_{1}=0.025$ g ms^−1^, $r_{2}=0.025$ g ms^−1^, $a_{01}=0.008$ cm^2^, $a_{02}=0.008$ cm^2^, L=0.56 cm, ρ=0.0013 g cm^−3^.

Parameter values of the vocal membrane were set as $m_{3}=\alpha \cdot 0.0016$ g, $d_{3}=\alpha \cdot 0.02$ cm, $\theta_{0}=0.01\pi$ rad, $k_{3}=\frac{1}{3}m_{3}d_{3}^{2}(2\pi f_{3}{)}^{2}$ g cm${,}^{2}$ ms^−^${,}^{2}$, $r_{3}=2\zeta \sqrt{\frac{1}{3}k_{3}m_{3}d_{3}^{2}}$ g cm${,}^{2}$ ms^−1^, where eigenfrequency and damping ratio of the vocal membrane were set as $f_{3}=300$ Hz and ζ=0.1. The length of the vocal membrane was scaled by a parameter α∈[1,5]. The differential equations were solved by using the *ode23* solver of MATLAB. To examine the effect of the vocal membranes, the vocal fold model having no vocal membranes (i.e*.* symmetric version of the Steinecke–Herzel model [30]) was also simulated in the same manner.

To detect the phonation threshold pressure, the model was simulated by varying the subglottal pressure in the range of $P_{s}\in \left[0\;\text{kPa},3\;\text{kPa}\right]$. First, for $P_{s}=0\;\text{kPa}$, the system was free-run for 1s from an initial condition of $(x_{1},{\dot{x}}_{1},x_{2},{\dot{x}}_{2},\theta ,\dot{\theta })=\left(0.1,0.1,0,0,\pi /20,0\right)$. After discarding a transient (duration of 0.5 s), the oscillation amplitude of the glottal flow u was recorded. The subglottal pressure was then increased by 30Pa and the next free-run was started from the final state of the previous free-run. The phonation onset pressure was detected at the point where the oscillation amplitude exceeded 0.00064 cm^3^ ms^−1^.

## 3. Results

### 3.1. Experiments

**Figure 1.**
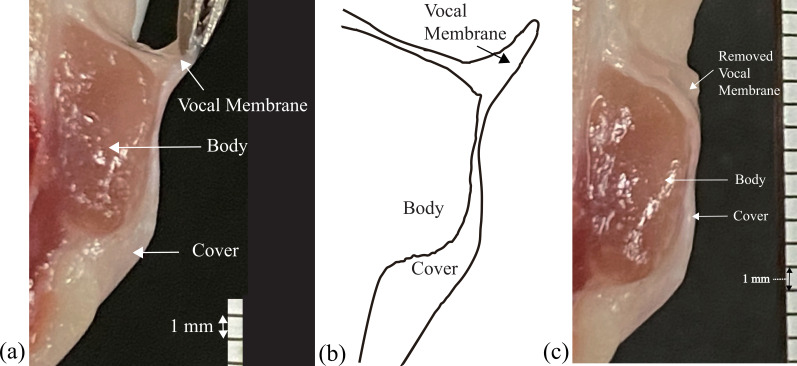
Frontal section of a hemi-larynx in one macaque. (a) The vocal membrane, located at the supero-medial portion of the vocal fold, is stretched by a pinset. (b) Line-traced image of (a), which highlights the vocal membrane as well as the body and cover layers of the vocal fold. (c) The vocal fold, from which the vocal membrane was removed.

First, laryngeal anatomy was inspected using another macaque individual no. 4, not used for the excised larynx experiments. As shown in figure 1a,b, the vocal membrane was clearly identified at the supero-medial part of the vocal fold. Such an intact vocal fold was studied as the condition (A), while the vocal fold, from which the vocal membranes were removed (see figure 1c), was examined as the control condition (B). For three macaque larynges, nos. 1, 2 and 3, the excised larynx experiments were carried out. Following the procedures explained in the method, three oscillation patterns were recorded: (A1) only the vocal membranes oscillated, (A2) the vocal folds and the vocal membranes co-oscillated, and (B) the vocal folds oscillated in the absence of the vocal membranes. Since no clear fundamental frequency was detected from the microphone signals of macaque no. 3, the observed oscillations were considered abnormal. Therefore, the data from macaque no. 3 were excluded from the analysis and shown only in the electronic supplementary material, figure S18.

**Figure 2.**
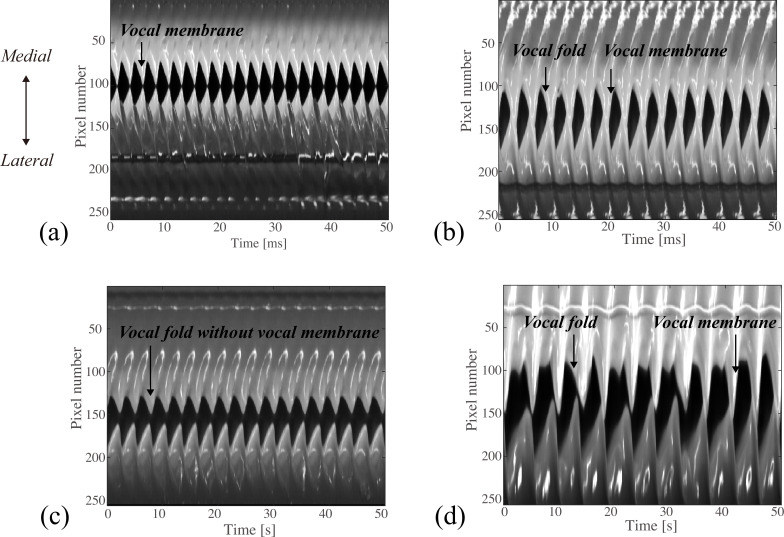
Kymograms extracted from the high-speed images of the electronic supplementary material, figures S2–S5. The horizontal axis represents time, whereas the vertical axis indicates the pixel location on the medial–lateral line. (a) The vocal membranes vibrate as the main oscillator, while the vocal folds do not much contribute to the oscillations. (b) The vocal folds and the vocal membranes co-vibrate simultaneously. (c) Dynamics of the vocal folds, from which the vocal membranes were removed. (d) Irregular oscillations of the vocal folds and the vocal membranes. (a–c) were recorded from macaque no. 1, while (d) was recorded from macaque no. 2.

From four high-speed movies measured from the excised larynx experiments (sequential images are displayed in the electronic supplementary material, figures S2–S5), kymograms were extracted in figure 2. Following [34], line images on the medial–lateral axis of the high-speed images (blue lines on the initial frames of the electronic supplementary material, figures S2–S5) were drawn in the order of time. The horizontal and vertical axes represent time and pixel location, respectively. The middle dark area corresponds to the glottal opening area, while the grey/white area indicates the surface of the vocal folds or the vocal membranes. Figure 2a–c are from the experiments on macaque no. 1, while figure 2d is from that on macaque no. 2. The vocal membranes were present in figure 2a,b,d, whereas they were removed in figure 2c. In figure 2a, the vocal membranes vibrate as the main oscillator, while the vocal folds do not appear to vibrate (i.e. oscillation pattern (A1); see the electronic supplementary material, figure S2 for detailed images). In figure 2b, in addition to the oscillations of the vocal membranes, the vocal folds oscillate as discernible during the closing phase (i.e. oscillation pattern (A2); see the electronic supplementary material, figure S3 for detailed images). In figure 2c, the vocal folds oscillate in the absence of the vocal membranes (i.e. oscillation pattern (B); see the electronic supplementary material, figure S4 for detailed images). In figure 2d, irregular oscillations of the vocal folds and the vocal membranes are clearly recognized (see the electronic supplementary material, figure S5 for detailed images). To provide a comprehensive view about the oscillatory patterns (A1), (A2) and (B), kymograms are presented for all recording data in the electronic supplementary material, figures S6–S8 (macaque no. 1) and figures S9–S11 (macaque no. 2). Spectrograms of the simultaneously recorded microphone signals are also presented in the electronic supplementary material, figures S12–S14 (macaque no. 1) and figures S15–S17 (macaque no. 2).

**Figure 3.**
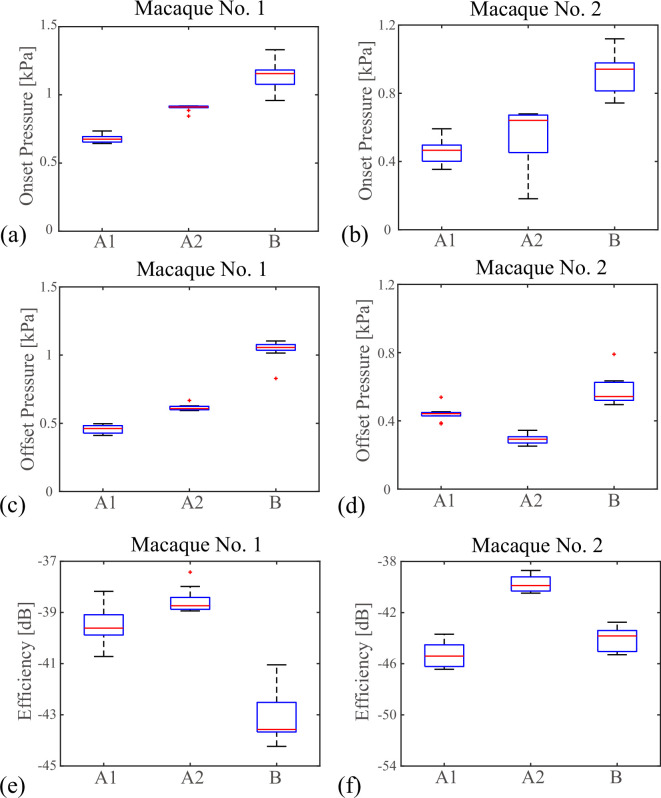
Comparison between the cases, in which (A1) only vocal membranes oscillate, (A2) vocal folds and vocal membranes co-oscillate, and (B) vocal folds oscillate in the absence of vocal membranes. Results for two macaque individuals (macaque no. 1: (a,c,e); macaque no. 2: (b,d,f)) are displayed. For each macaque larynx, the quantities were computed from 10 datasets and drawn with box plots. (a,b) Phonation onset pressure detected by the subglottal pressure signal. (c,d) Phonation offset pressure detected by the subglottal pressure signal. (e,f) Vocal efficiency computed from the subglottal pressure and the sound pressure level.

For each of the three oscillation patterns (A1), (A2) and (B), the phonation onset and offset pressures and the vocal efficiencies were measured and the results are shown in figure 3. In figure 3a,b, the phonation onset pressures are compared between the three patterns. In both macaque individuals (nos. 1 and 2), the highest onset pressure was observed when the vocal membranes were removed from the vocal folds. Namely, patterns (A1) and (A2) showed significantly lower pressures than that of pattern (B) (macaque no. 1: *F*_2,27_ = 217.0, $p=2.3\;\times \;10^{-17}$; $p_{\;A1<A2}=5.4\;\times \;10^{-10}$, $p_{\;A1<B}=2.3\;\times \;10^{-18}$, $p_{\;A2<B}=5.9\;\times \;10^{-11}$; macaque no. 2: *F*_2,27_  = 32.8 , $p=6.0\;\times \;10^{-8}$; $p_{\;A1<A2}=0.38$, $p_{\;A1<B}=1.1\;\times \;10^{-7}$, $p_{\;A2<B}=3.3\;\times \;10^{-6}$). Similar tendency was confirmed for the phonation offset pressure of figure 3c,d, where the highest offset pressure was again observed for pattern (B) in the two macaque individuals (nos. 1 and 2). In macaque no. 1, the offset pressure of pattern (A1) was lower than that of pattern (A2) but this relationship was reversed in macaque no. 2 (macaque no. 1: *F*_2,27 _= 1321.2, $p=1.2\;\times \;10^{-27}$; $p_{\;A1<A2}=8.6\;\times \;10^{-13}$, $p_{\;A1<B}<10^{-35}$, $p_{\;A2<B}=2.1\;\times \;10^{-34}$; macaque no. 2: *F*_2,27 _= 57.4, $p=1.9\;\times \;10^{-10}$; $p_{\;A1>A2}=1.6\;\times \;10^{-5}$, $p_{\;A1<B}=7.3\;\times \;10^{-5}$, $p_{\;A2<B}=9.5\;\times \;10^{-11}$). As shown in figure 3e,f, the vocal efficiency was highest in pattern (A2) in the two macaque individuals (macaque no. 1: *F*_2,27 _= 94.8, $p=6.2\;\times \;10^{-13}$; $p_{\;A1<A2}=0.015$, $p_{\;A1>B}=3.2\;\times \;10^{-10}$, $p_{\;A2>B}=8.8\;\times \;10^{-13}$; macaque no. 2:  *F*_2,27 _= 7.95, $p=1.9\;\times \;10^{-3}$; $p_{\;A1<A2}=0.008$, $p_{\;A1<B}=0.93$, $p_{\;A2>B}=0.0034$).

**Figure 4.**
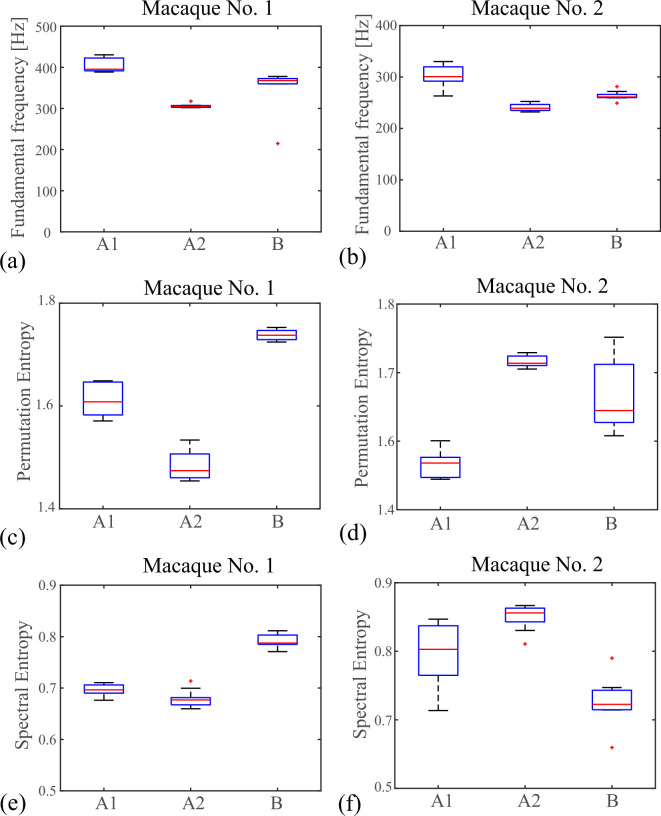
Complexity analysis of the three oscillation patterns (A1), (A2) and (B). Microphone signals were analysed for two macaque individuals (macaque no. 1: (a,c,e); macaque no. 2: (b,d,f)). The fundamental frequencies $f_{o}$, permutation entropies and spectral entropies were computed in (a,b), (c,d) and (e,f), respectively. For each oscillation pattern, the quantities were obtained from 10 datasets and drawn with box plots.

Next, the fundamental frequencies $f_{o}$ were computed from the microphone signals and are drawn in figure 4a,b. In both macaque individuals (nos. 1 and 2), $f_{o}$ was highest in pattern (A1) and lowest in pattern (A2) (macaque no. 1: *F*_2,22_= 190.4, $p=1.3\;\times \;10^{-14}$; $p_{\;A1>A2}=4.1\;\times \;10^{-15}$, $p_{\;A1>B}=4.2\;\times \;10^{-5}$, $p_{\;A2<B}=2.2\;\times \;10^{-9}$; macaque no. 2: *F*_2,26_ = 55.4, $p=4.22\;\times \;10^{-10}$; $p_{\;A1>A2}=2.6\;\times \;10^{-10}$, $p_{\;A1>B}=3.1\;\times \;10^{-6}$, $p_{\;A2<B}=0.002$).

We note that the observed quantities are comparable to those of previous studies on excised larynx experiments of primate species (squirrel monkey (*S. boliviensis*): phonation onset pressure in the range of 1 kPa, 3 kPa [6]; common marmosets (*C. jacchus*): phonation onset pressure in the range of 2.05±0.37 kPa (infant) and 0.62±0.13 kPa (adult), fundamental frequency in the range of 0.5 kHz, 12 kHz (infant) and 2 kHz, 6 kHz (adult) and mechanical efficiency in the range of −67 dB, −60 dB (infant) and −60 dB, −52 dB (adult) [7]; coo calls of Japanese macaques (*Macaca fuscata*): fundamental frequency in the range of 585±74.1 Hz [35]; normal vocalization of rhesus macaques (*M. mulatta*): fundamental frequency in the range of 300 Hz, 500 Hz, phonation onset pressure in the range of 0.5 kPa, 1.2 kPa and vocal efficiency in the range of −57 dB, −42 dB [36]). This confirms the plausibility of our experiments.

Next, as a measure of complexity, the permutation entropies as well as the spectral entropies were computed for the microphone signals. The embedding dimension and the time delay were set to d=3 and $\tau =1/\{4(d-1)f_{o}\}$, respectively [23]. The three oscillation patterns (A1), (A2) and (B) are compared in figure 4c–f. In macaque no. 1, the permutation entropies were significantly lower in pattern (A2) than in patterns (A1) and (B) (*F*_2,24_= 219.2, $p=3.8\;\times \;10^{-16}$; $p_{\;A1>A2}=4.4\;\times \;10^{-10}$, $p_{\;A1<B}=7.9\;\times \;10^{-10}$, $p_{\;A2<B}=5.0\;\times \;10^{-17}$). These results are consistent with those of the spectral entropies (*F*_2,24_= 244.0, $p=1.1\;\times \;10^{-16}$; $p_{\;A1>A2}=0.005$, $p_{\;A1<B}=1.3\;\times \;10^{-14}$, $p_{\;A2<B}=7.8\;\times \;10^{-17}$), indicating that co-oscillations of the vocal folds and the vocal membranes do not exhibit very complex patterns in macaque no. 1. Indeed, the corresponding kymograms showed periodic oscillatory patterns (see figure 2b and the electronic supplementary material, figure S7).

In macaque no. 2, on the other hand, the permutation entropies as well as the spectral entropies were significantly higher in pattern (A2) (permutation entropy: *F*_2,24_= 43.7, $p=9.9\;\times \;10^{-9}$; $p_{\;A1<A2}=6.8\;\times \;10^{-9}$, $p_{\;A1<B}=1.2\;\times \;10^{-5}$, $p_{\;A2>B}=0.01$; spectral entropy: *F*_2,24_= 29.2, $p=3.7\;\times \;10^{-7}$; $p_{\;A1<A2}=0.01$, $p_{\;A1>B}=4.8\times 10^{-4}$, $p_{\;A2>B}=2.2\times 10^{-7}$). This indicates that the oscillations tend to be more complex as the vocal folds and the vocal membranes oscillate together in macaque no. 2. These results agree with the corresponding kymograms, which show irregular oscillatory patterns (see figure 2d and the electronic supplementary material, figure S10). This suggests that, depending upon the phonation condition and the individual characteristics, co-oscillations of the vocal folds and the vocal membranes may potentially lead to voice instability.

### 3.2. Computational model

**Figure 5.**
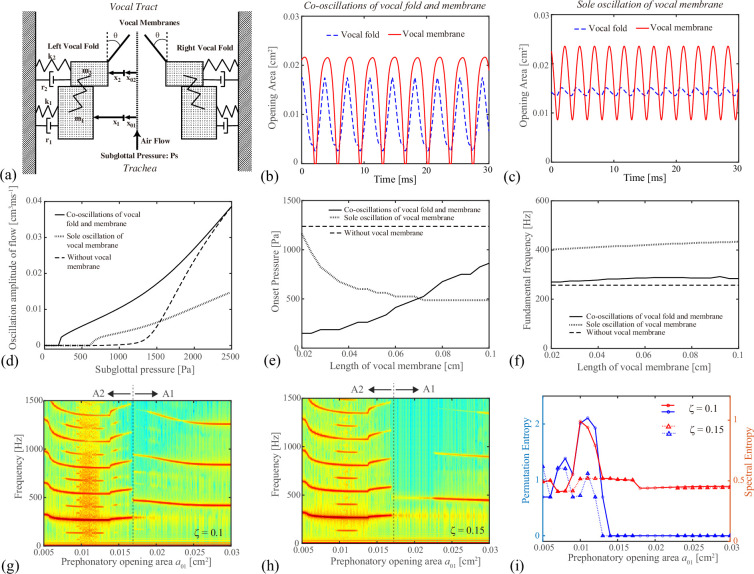
Results of the computational model. (a) Schematic illustration of the vocal membrane model. The vocal folds are represented by lower and upper masses $m_{1}$, $m_{2}$, while the vocal membrane is attached to the upper mass as a reed-like plate. (b,c) Time traces of the opening areas of the vocal folds (dashed blue line) and the vocal membranes (red solid line) with $P_{s}=2.4$ kPa, $d_{3}=0.04$ cm, $a_{01}=0.008$ cm^2^ for (b), $P_{s}=2.4$ kPa, $d_{3}=0.04$ cm, $a_{01}=0.08$ cm^2^ for (c). (d) Dependence of the oscillation amplitude of the flow U on the subglottal pressure $P_{s}$ in the case of $d_{3}=0.04$ cm. (e) Dependence of the phonation onset pressure on the length $d_{3}$ of the vocal membrane. (f) Dependence of the fundamental frequency on the length $d_{3}$ of the vocal membrane. In (d–f), three oscillation patterns (solid line: co-oscillations of vocal folds and membranes, dashed line: sole oscillation of vocal membranes, dotted line: model without vocal membranes) are compared. (g,h) Bifurcation diagrams of the model, in which the prephonatory opening area of the lower mass was changed from $a_{01}=0.005$ to 0.03 cm^2^. The damping ratio was set to ζ=0.1 in (g) and ζ=0.15 in (h). Other parameter values were fixed to $P_{s}=2.5$ kPa, $d_{3}=0.04$ cm. (i) Permutation entropy (blue) and spectral entropy (red) corresponding to panels (g: solid lines with circles) and (h: dotted lines with triangles).

To reproduce our experiments, the mathematical model of the vocal membrane was simulated (see figure 5a). First, the length of the vocal membrane, the prephonatory opening area of the lower mass and the subglottal pressure were set to their default values of $d_{3}=0.04$ cm (i.e. α=2), $a_{01}=0.008$ cm^2^ and $P_{s}=2.4$ kPa, respectively. Figure 5b shows time traces of the opening areas of the vocal folds $a_{\text{vf}}$ and the vocal membranes $a_{\text{vm}}$. Movements of the vocal membranes were phase-delayed from those of the vocal folds during the closing phase, reproducing the experimentally observed oscillation pattern (A2) (see figure 2b). Figure 5c, on the other hand, shows the case, in which the vocal folds were abducted by setting the prephonatory opening area of the lower mass to $a_{01}=0.08$ cm^2^. As expected, only the vocal membranes vibrated, while the vocal folds exhibited only a slight movement. This corresponds to the experimentally observed oscillation pattern (A1) (see figure 2a). It should be noted that, in the experiment, the left and right vocal membranes touched each other at the closing phases. By contrast, the simulated vocal membranes were more distant from each other, because the left and right vocal folds, on which the vocal membranes were located, oscillated only slightly and were apart from each other. Consequently, the time trace of the glottal opening area showed a harmonic waveform, which gave rise to a strong fundamental frequency component with relatively weak higher harmonics (see figure 5g,h).

Next, for the two oscillation patterns, the phonation onset was detected. In figure 5d, the oscillation amplitude of the glottal flow was drawn by increasing the subglottal pressure. The phonation onset point, at which the oscillation amplitude became larger than 0.00064 cm^3^ ms^−1^, was detected at 640 and 225Pa for oscillation patterns (A1) and (A2), respectively. To examine the effect of the vocal membranes, the vocal fold model having no vocal membranes (i.e*.* pattern (B)) was also simulated and its onset pressure was detected at 1463Pa. Compared with the model without vocal membranes, the phonation onset pressure was significantly lowered in the presence of the vocal membranes.

One of the key parameters that may influence the onset pressure is the length of the vocal membrane $d_{3}$. To see this influence, the phonation onset pressure as well as the fundamental frequency $f_{o}$ was calculated by varying the length from $d_{3}=0.02$ to 0.1 cm in figure 5e,f. In the case of oscillation pattern (A2), the onset pressure ranged between 150 and 862Pa ($f_{o}\;\approx \;280.9\pm 7.6$ Hz). In the case of oscillation pattern (A1), the onset pressure decreased monotonously from 1162 to 488Pa ($f_{o}\;\approx \;422\pm 12$ Hz) as the membrane length was increased to $d_{3}=0.1$ cm. In the whole range $d_{3}\in \left[0.02\;\text{cm},0.1\;\text{cm}\right]$, the phonation onset pressure was lower for the model with vocal membranes compared with the one without vocal membranes (i.e. 1238Pa). These results are consistent with the theoretical study based on the static vocal membrane model [5] (i.e. angle of the membrane does not change in time), which states that the vocal membrane can lower the phonation onset pressure. Additional finding here is that this statement is also valid in the case that the vocal membranes move dynamically in time.

In the present model, the prephonatory opening area of the lower mass $a_{01}$ plays a key role in changing the oscillation pattern from (A2) to (A1). To study the process of their transition, bifurcation diagrams were drawn in a range of $a_{01}\in \left[0.005\;{\text{cm}}^{2},0.03\;{\text{cm}}^{2}\right]$ in figure 5g,h. The damping ratio was set to the default value of ζ=0.1 in figure 5g and to an increased value of ζ=0.15 in figure 5h. The subglottal pressure was fixed to $P_{s}=2.5$ kPa. In figure 5g, a small opening area ($a_{01}=0.005$ cm^2^) displayed the oscillation pattern (A2) with a low fundamental frequency ($f_{o}\;\approx \;300$ Hz) accompanied by its higher harmonics. As the opening area was increased, subharmonic bifurcation took place, which led to chaotic oscillations around $a_{01}=0.011$ cm^2^. As the opening area was further increased, periodic oscillation of pattern (A2) was recovered and around $a_{01}=0.017$ cm^2^ (dashed line) it switched to the oscillation pattern (A1) with a high fundamental frequency ($f_{o}\;\approx \;450$ Hz). In figure 5h, a similar transition from (A2) to (A1) was observed except for the fact that the chaotic oscillatory regime disappeared.

To quantify the complexity of the oscillation patterns, the permutation entropies [20,21] and the spectral entropies [22] were computed for the corresponding glottal waveforms in figure 5i. As expected from the bifurcation diagram of figure 5g, both quantities showed peaks around $a_{01}=0.011$ cm^2^, where the spectral structure became chaotic. Such peaks disappeared in the complexity measures corresponding to the bifurcation diagram of figure 5h. Thus, the situation of figure 5h may represent the periodic kymograms observed in macaque no. 1 in figure 2a,b. On the other hand, the situation of figure 5g may potentially correspond to the irregular kymogram observed in macaque no. 2 in figure 2d, although further investigation is needed to judge whether the experimentally observed irregularity is truly owing to chaos.

## 4. Conclusions and discussion

To study the effect of the vocal membranes on the vocalization of rhesus macaques, excised larynx experiments were carried out. The oscillation properties were compared between larynges with and without vocal membranes. The experiments indicated that, in the presence of the vocal membranes, two oscillation patterns existed: (A1) only the vocal membranes oscillated, and (A2) the vocal folds and the membranes co-oscillated. Compared with pattern (B), in which the vocal folds oscillated in the absence of the vocal membranes, the phonation onset and offset pressures were significantly lowered by the involvement of the vocal membranes. This provides an experimental confirmation of the theoretical study [5], which predicted that the vocal membranes can lower the phonation threshold pressure, thus increasing the vocal efficiency. In our experiments, the vocal efficiency was indeed increased when the vocal folds and the vocal membranes co-oscillated.

Our experiments also showed that the fundamental frequency increased when only the vocal membranes oscillated. The increased fundamental frequency might be owing to the high eigenfrequency inherent in the vocal membranes.

Concerning oscillation pattern (A2), the kymogram indicated that the vocal membranes synchronized with the vocal folds with some phase delay. Such a delay was confirmed *in vivo* in macaques and chimpanzees [4]. The delayed dynamics of the vocal membranes may enhance convergent-divergent motions of the vocal folds, which enable an efficient energy transfer from the airflow to the tissue vibrations [37]. Consequently, the phonation threshold pressures might have been lowered. One of the physiological characteristics of macaques is that the vocal fold cover layer is thinner than that of humans and the lamina propria is dense in the fibrous tissue [38,39]. Upon such a thin and hard cover, the mucosal waves may not be formed so strongly. To compensate for such weak mucosal wave propagation, the vocal membranes could be of good help for strengthening the laryngeal oscillations in macaque vocalizations.

In our experiments, most excised larynges showed periodic vibrations. One exception was condition (A2) of macaque no. 2, which exhibited irregular oscillatory patterns. To quantify the level of complexity in such oscillations, the permutation entropy and the spectral entropy were computed. It was found that the complexity was generally higher in oscillation pattern (A2) than in oscillation patterns (A1) and (B) in macaque no. 2. Note that, while elevated permutation entropy suggests increased dynamical complexity, it does not by itself confirm the presence of deterministic chaos. By contrast, in macaque no. 1, oscillation pattern (A2) was rather periodic and the complexity was lower than the other oscillation patterns. This suggests that interaction between the vocal folds and the vocal membranes does not necessarily lead to voice instability. Whether co-oscillations of the vocal folds and membranes produce complex irregular dynamics may depend upon the phonation condition and the individual characteristics. We also note that, when voice instability is observed, it is not straightforward to identify the exact cause, because other factors such as left–right asymmetry [30], anterior–posterior modes [40], excessively high subglottal pressure [41], etc*.* can also induce chaos. It should be carefully examined whether the experimentally observed irregular oscillations of the vocal folds were truly owing to the vocal membranes.

To reproduce our experiment, a mathematical model was further simulated. The vocal membrane model showed that the membranes can substantially lower the phonation threshold pressure, compared with the model having no vocal membranes. By changing the prephonatory opening area of the lower part of the vocal folds, the oscillation pattern could be switched between patterns (A1) and (A2). Depending upon the damping ratio, both chaotic and non-chaotic transitions were observed. The non-chaotic transition may represent the experimental situation of macaque no. 1, which displayed periodic oscillatory patterns. The chaotic transition might be potentially related to the irregular oscillatory patterns (A2) observed in macaque no. 2.

From the viewpoint of animal bioacoustics, the present study suggests that the vocal membranes can support the vocalization by lowering the phonation threshold pressure, thereby making the vocal fold oscillations more efficient. Such a property can be advantageous for animals when producing loud calls with less power from the lungs.

For a more comprehensive understanding of the functions of the vocal membranes, further investigations are needed. In particular, our study is based only on two larynges from a single macaque species. Our findings are therefore limited and cannot be over-generalized. More larynges should be examined in the future to validate our study. Considering the difference from other primate species, the vocal membrane should be varied in length, thickness and its location relative to the vocal folds, and, moreover, the vocal fold itself has a variability in anatomy [4]. In addition, the laryngeal air sac, which is connected to a laryngeal region in some clades of non-human primates [3,42,43], may also contribute to the phonation dynamics [44,45]. Examination of other species should clarify the dependence of the phonation threshold pressures as well as the vocal efficiencies on such laryngeal variabilities. Finally, vocalizations are known to be mechanically inefficient compared with body movement or other physical activities in animals [46]. It should be thus questioned whether realization of efficient vocalizations can be of significant importance when viewed in competition with other energy needs. Such an issue should be addressed in a future study.

## Data Availability

The data supporting this article are available at Dryad [47]. Supplementary material is available online [48].
